# Transversal Halide Motion Intensifies Band‐To‐Band Transitions in Halide Perovskites

**DOI:** 10.1002/advs.202200706

**Published:** 2022-04-04

**Authors:** Christian Gehrmann, Sebastián Caicedo‐Dávila, Xiangzhou Zhu, David A. Egger

**Affiliations:** ^1^ Department of Physics Technical University of Munich James‐Franck‐Straße 1 Garching 85748 Germany

**Keywords:** disorder, halide perovskites, molecular dynamics, optoelectronic properties, urbach energy

## Abstract

Despite their puzzling vibrational characteristics that include strong signatures of anharmonicity and thermal disorder already around room temperature, halide perovskites (HaPs) exhibit favorable optoelectronic properties for applications in photovoltaics and beyond. Whether these vibrational properties are advantageous or detrimental to their optoelectronic properties remains, however, an important open question. Here, this issue is addressed by investigation of the finite‐temperature optoelectronic properties in the prototypical cubic CsPbBr_3_, using first‐principles molecular dynamics based on density‐functional theory. It is shown that the dynamic flexibility associated with HaPs enables the so‐called *transversality*, which manifests as a preference for large halide displacements perpendicular to the Pb‐Br‐Pb bonding axis. The authors find that transversality is concurrent with vibrational anharmonicity and leads to a rapid rise in the joint density of states, which is favorable for photovoltaics since this implies sharp optical absorption profiles. These findings are contrasted to the case of PbTe, a material that shares several key properties with CsPbBr_3_ but cannot exhibit any transversality and, hence, is found to exhibit much wider band‐edge distributions. The authors conclude that the dynamic structural flexibility in HaPs and their unusual vibrational characteristics might not just be a mere coincidence, but play active roles in establishing their favorable optoelectronic properties.

## Graphical abstract

Halide perovskites are materials that exhibit anharmonic vibrational characteristics and intriguing atomic motions at finite temperature. Here, using first‐principles molecular dynamics for the prototypical compound CsPbBr_3_, it is found that transversal halide motions lead to intensified band‐to‐band transitions, which is an important feature for sharp optical absorption profiles and efficient capture of sunlight.

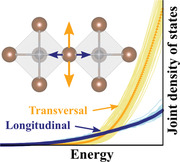

## 1 Introduction

Halide perovskites (HaPs) are semiconducting materials with an enormous potential for various technological applications, perhaps most notably for photovoltaics.^[^ ^1^, ^2^, ^3^, ^4^ ^]^ Significant interests in these systems are motivated by their favorable optoelectronic properties and the simultaneous availability of low‐cost synthesis and fabrication routes.^[^ ^5^, ^6^, ^7^, ^8^ ^]^ Among their many outstanding physical and chemical characteristics, the structural dynamical properties of HaPs are especially intriguing.^[^ ^9^, ^10^, ^11^ ^]^ In particular, significant vibrational anharmonicities have been detected in these and related systems already at room temperature.^[^ ^12^, ^13^, ^14^, ^15^, ^16^, ^17^, ^18^, ^19^, ^20^, ^21^ ^]^ The unusual lattice vibrational properties of HaPs have already been shown to influence some key optoelectronic quantities including the band‐gap energy,^[^ ^22^ ^]^ charge‐carrier mobilities,^[^ ^23^, ^24^, ^25^ ^]^ defect energetics,^[^ ^26^, ^27^, ^28^, ^29^, ^30^, ^31^ ^]^ the Urbach energy,^[^ ^32^, ^33^ ^]^ carrier recombination rates and ion migration barriers,^[^ ^34^, ^35^, ^36^, ^37^ ^]^ It is an important open question to what extent the peculiar lattice vibrational properties of HaPs influence their optoelectronic properties.^[^ ^37^ ^]^ Since vibrational anharmonicities are known to occur in other optoelectronic materials,^[^ ^38^, ^39^, ^40^, ^41^, ^42^, ^43^, ^44^, ^45^, ^46^, ^47^ ^]^ addressing this question is important because it will support the design of alternative material systems with similarly favorable or improved optoelectronic properties.

In this work, we perform first‐principles molecular dynamics (MD) simulations to investigate the origins and consequences of the unusual lattice vibrational properties of HaPs. Specifically, the goal of our work is to investigate the connections between the lattice vibrations and the optical absorption profile of HaPs. In characterizing the vibrational features of the prototypical cubic CsPbBr_3_ we highlight a distinctive property—which we call *transversality*—that showcases the high degree of dynamic structural flexibility in HaPs. Transversality is shown to derive naturally from the octahedral arrangement of halide ions in the perovskite lattice and to coincide with large vibrational displacements and anharmonicities in the system. Importantly, the dynamic structural flexibility of HaPs as expressed in transversality is found to have a strong effect on disorder correlations that are important for optoelectronic properties including finite‐temperature band‐gap energy distributions and possible band‐to‐band transitions. We contrast the findings for CsPbBr_3_ to the case of PbTe, which shares several important characteristics with prototypical HaPs but, due to its rocksalt structure, cannot exhibit any transversality, in order to demonstrate that absence of this feature is detrimental to optoelectronic properties. Our results suggests that the peculiar lattice vibrational properties of HaPs might not just be a mere coincidence, but rather could potentially play an active role in determining the favorable optoelectronic properties of these materials.

## 2 Results and Discussion

**Figure 1:**
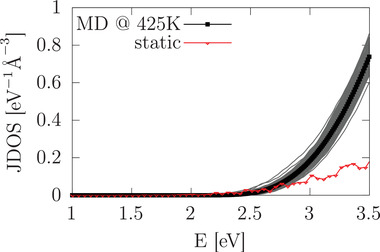
Joint density of states (JDOS) of CsPbBr_3_, calculated for a static cubic structure (red) and from molecular dynamics (MD) at 425 K (black). Thin‐black lines correspond to the JDOS of individual MD snapshots and the thick‐dotted line to the their average.

We investigate the protoypical HaP material CsPbBr_3_, which is known to exhibit, on average, a cubic‐symmetry structure of corner‐sharing octahedra and void‐filling A‐site cations at temperatures above ≈400 K.^[^ ^48^, ^49^, ^50^ ^]^ We consider CsPbBr_3_ in its cubic phase because this is the regime where the vibrational characteristics are particularly interesting, for example, previous work found that a broad central peak emerges in the Raman spectrum of cubic CsPbBr_3_ due to local polar fluctuations. ^[^ ^14^ ^]^ Performing MD calculations based on density‐functional theory (DFT) at a temperature of 425 K (see Methods Section) allows for depicting the finite‐temperature, real‐time structure of cubic CsPbBr_3_, including all vibrations excited at that temperature and a description of the appearing vibrational anharmonicity to all orders. **Figure** **1** shows the joint density of states (JDOS) of CsPbBr_3_, calculated either for a static cubic structure or for snapshots of instantaneous structures that were recorded along the MD trajectory at 425 K. The JDOS connects the electronic structure of a system to its optical absorption, because it quantifies the possible band‐to‐band transitions in the single‐particle band‐structure assuming constant transition matrix elements.^[^ ^51^, ^52^ ^]^ Clearly, the two scenarios yield vastly different results: first, the onset of the JDOS on the energy axis differs by ≈0.5 eV and, second, the JDOS rises much more rapidly in the 425 K data. The first finding is related to the fact that the band‐gap energy of the averaged, cubic‐symmetry structure calculated in a static DFT calculation differs substantially (by ≈0.7 eV) from the average band‐gap energy calculated as a time‐average of the MD calculation at 425 K, as has been discussed in previous work.^[^ ^88^ ^]^ The second finding, that is, that the JDOS rises more rapidly in the 425 K MD than in the static DFT calculation, is particularly interesting because previous work has highlighted that already static HaPs feature a JDOS that is steeper close to the absorption edge when compared to other semiconductors.^[^ ^53^, ^54^, ^55^ ^]^ Here, we find that the JDOS is intensified in the MD at 425 K compared to the result of the static calculation. Taken together, these findings demonstrate that vibrational features occurring in cubic CsPbBr_3_ at 425 K strongly impact the JDOS, and we will therefore investigate which patterns in the finite‐temperature atomic dynamics are responsible for it.

**Figure 2:**
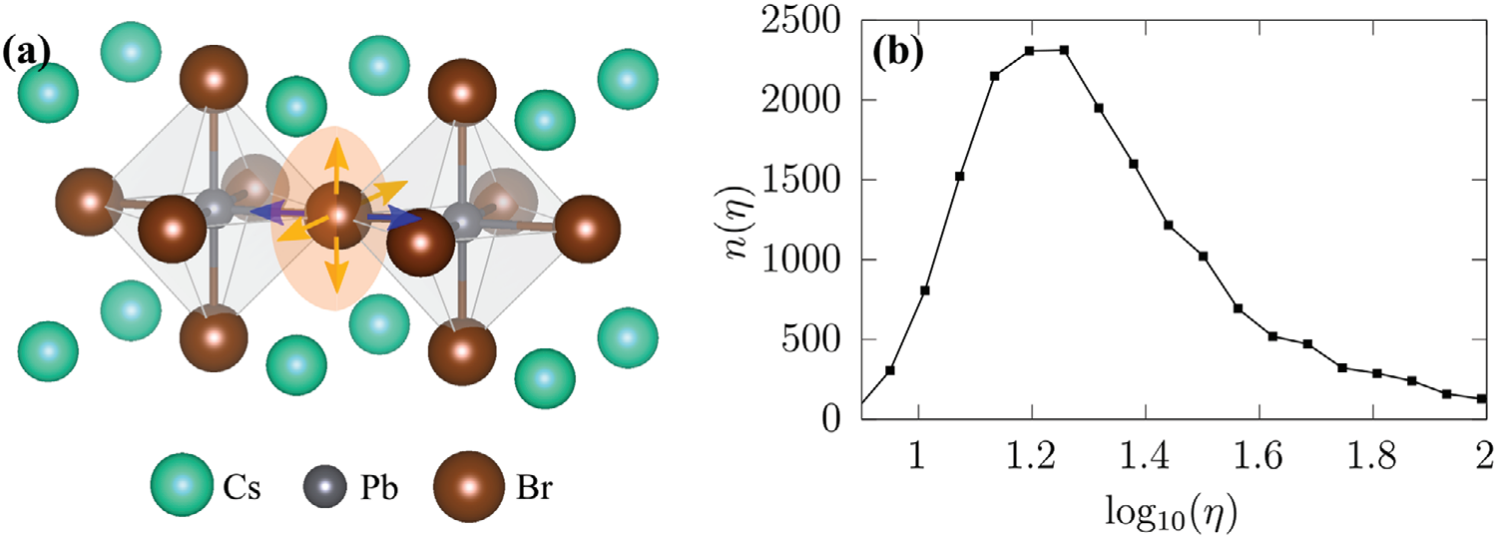
a) Schematic structural representation of cubic CsPbBr_3_ and the displacements of Br ions at finite‐temperature: longitudinal components occur in parallel to the Pb–Br–Pb bonding axis (blue arrows) and transversal components perpendicular to it (yellow arrows expanding the light‐orange plane). b) Histogram of transversality, η, as defined in Equation (1), calculated along the MD trajectory at 425 K. Note that this is shown as a semi‐log plot.

Since electronic states close to the band edges of HaPs in general, and CsPbBr_3_ in particular, are formed by the lead and halide atomic orbitals, we focus on the dynamics in the Pb–Br–Pb network and motions of halides therein, and stipulate to describe the finite‐temperature Br displacements occurring in the MD by two directional components: either Br atoms move along the Pb–Br–Pb bond axis (blue line in **Figure** **2**a) or on the 2D plane that is perpendicular to that axis (light‐orange plane in Figure 2a). Thus, any Br displacement occurring at finite temperature is characterized by its longitudinal (along the Pb–Br–Pb bond axis) and transversal components (perpendicular to the Pb–Br–Pb bond axis).

For each structure occurring along the MD trajectory we hence define a transversality, η, as follows

(1) $$\eta =\frac{1}{N_{\mathrm{Br}}}\sum_{i=1}^{N_{\mathrm{Br}}}\frac{d_{i}^{\mathrm{transv}}}{d_{i}^{\mathrm{longi}}}$$

where *N*_Br_ is the number of Br atoms in the supercell and $d_{i}^{\mathrm{transv}/\mathrm{longi}}$ are the norms of the transversal / longitudinal displacement components of the *i*th Br ion. We present additional means of comparing the occurrence of transversal and longitudinal displacements in the Supporting Information. Calculating the histogram of η along the MD trajectory (see Figure 2b) reveals that CsPbBr_3_ in its cubic structure features large transversal Br displacements: the histogram peaks at η ≈ 20, with a significant tail at high values of η, up to η ≈ 100, and quickly decreases to zero when approaching η = 1. This indicates that the movements of Br ions are very much favored on the 2D‐planes perpendicular to Pb–Br–Pb bond axis compared to movements along it, which suggests a peculiar directionality in the finite‐temperature dynamics of CsPbBr_3_ despite the fact that, on average, it exhibits a cubic symmetry. Interestingly, the appearance of transversality is closely connected to the rotations of the PbBr_6_ octahedra (see Supporting Information), which are a known generic feature of the finite‐temperature dynamics of HaPs.^[^ ^56^, ^57^, ^58^, ^59^ ^]^ In the Supporting Information, we also show that the transversal Br displacements are concurrent with lattice‐vibrational anharmoncity in CsPbBr_3_.

**Figure 3:**
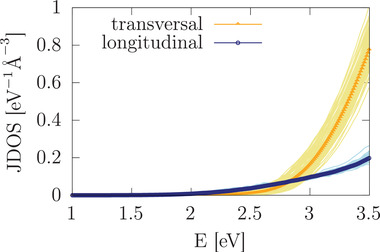
MD‐calculated JDOS of CsPbBr_3_ at 425 K corresponding to Br motions that occur either along longitudinal (blue) or transversal (yellow) directions. Thin lines correspond to data of individual snapshots and thick‐dotted lines to their average.

The large degree of transversality in the Br ion dynamics signifies an enormous dynamic structural flexibility of HaPs because Br ions that participate in covalent bonding with two adjacent Pb ions are actually favorably displaced away from the Pb–Br–Pb bonding axis. The occurrence of this property motivates an investigation into its origin and implications for other important characteristics of CsPbBr_3_. We therefore calculate the JDOS again from MD of CsPbBr_3_ at 425 K, but now separately for longitudinal and transversal displacements of Br atoms (see **Figure** **3**). Interestingly, this procedure finds results that essentially mimic what we have reported above for the JDOS in case of the static lattice and at 425 K. Most importantly, the transversal version of the JDOS rises much faster than the longitudinal one, such that there is a clear connection between the occurrence of transversality and a rapid increase in JDOS. This finding illustrates a remarkable aspect in how the dynamic structural flexibility of HaPs determines their optoelectronic properties, namely such that transversal Br motion intensifies the possible band‐to‐band transitions in CsPbBr_3_.

**Figure 4:**
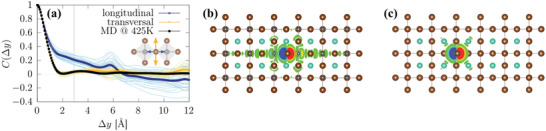
a) Autocorrelation function of the disorder potential, *C*(Δ*y*), that is induced by the Br motions along longitudinal (blue) and transversal (yellow) directions in CsPbBr_3_ at 425 K. The thin lines correspond to individual snapshots, *C*_*i*_(Δ*y*) in Equation (3), and the thick‐dotted lines to their average. *C*(Δ*y*) according to the full set of displacements occurring in the 425 K trajectory is also shown (black line). The inset shows two adjacent PbBr_6_ octahedra and depicts the Br displacement directions, and the vertical gray line indicates the nominal Pb–Br bonding distance. Isosurface representation of the calculated charge‐density response to a Br displacement along b) longitudinal direction and c) transversal direction in a supercell of CsPbBr_3_. Isosurfaces are shown for changes above 8.1 × 10^−3^ *e*Å ^−3^ (*e* being the electron charge).

To investigate the origin of dynamic structural flexibility and large degree of transversality in CsPbBr_3_, we analyze the disorder potential generated by the finite‐temperature ion dynamics. Specifically, we compute the autocorrelation function of the disorder potential, *C*(Δ*y*) (see Methods Section), for which we have previously found that it is dynamically shortened and approaches a value of zero on length scales on the order of one Pb–X bond.^[^ ^33^ ^]^ **Figure** **4**a shows *C*(Δ*y*) separately for the longitudinal and transversal components of Br displacements in CsPbBr_3_ at 425 K (see Methods Section), in addition to the result from a full MD calculation. While the longitudinal components alone would lead to a long‐ranged response, clearly the transversal Br displacements are responsible for the short‐ranged nature of the disorder potential in CsPbBr_3_ at finite temperature. Such a short‐ranged disorder potential is known to be concurrent with narrower band‐edge distributions that imply small values for the Urbach energy.^[^ ^33^, ^60^ ^]^ Keeping in mind that other effects are of course also potentially important for determining the Urbach energy of HaPs,^[^ ^61^ ^]^ the finding shows that the transversality in the Br dynamics is an important factor that leads to a rapid rise of the JDOS and sharp optical absorption.

The reason for the transversality in Br displacements and the short‐ranged nature of the disorder potential can be found in the very peculiar way the HaP system responds to changes in its atomic configurations. To illustrate this, we calculated the charge‐density response to specific atomic displacements in an otherwise static supercell of CsPbBr_3_ using DFT (see Methods Section for details). Displacing a Br ion longitudinally leads to a response in the charge density that is clearly long‐ranged and spans several unit cells (Figure 4b). In sharp contrast to this scenario, displacing a Br ion transversely leads to a response in the charge density that is clearly short‐ranged and essentially confined to a single unit cell (Figure 4c). This qualitative difference in the charge‐density response comparing transversal and longitudinal Br displacements is related to the resonant bonding mechanism^[^ ^62^, ^63^ ^]^ that has been extensively discussed in the literature on HaPs^[^ ^19^, ^33^, ^64^ ^]^ and other materials:^[^ ^65^, ^66^, ^67^, ^68^ ^]^ along the Pb–Br–Pb bond axes, Br‐4p_*x*_ and Pb‐6s σ‐interactions give rise to the valence band and a network of resonant bonds.^[^ ^69^, ^70^ ^]^ Displacing an atom along the direction of orbital hybridization leads to changes in the charge density that “resonate” throughout the network: when the symmetry of the network is broken by such a displacement, degeneracies of orbital configurations are lifted, the orbital occupations thus modulated, and a long‐ranged response in the charge density must follow (*cf*., Figure 4b). In contrast, any transversal displacement cannot influence the resonant bonding network, since by its definition the transversal plane is orthogonal to the direction of the network, as are the Br‐p_*y*_ and Br‐p_*z*_ states which do not participate in the resonant bonding. Therefore, the transversal Br displacements essentially do not perturb the resonant network and, hence, only lead to short‐ranged changes in the charge density (*cf*., Figure 4c). It is for this reason that transversal Br displacements are highly favored energetically (see Supporting Information) over the longitudinal ones and, hence, occur with a much larger likelihood.

**Figure 5:**
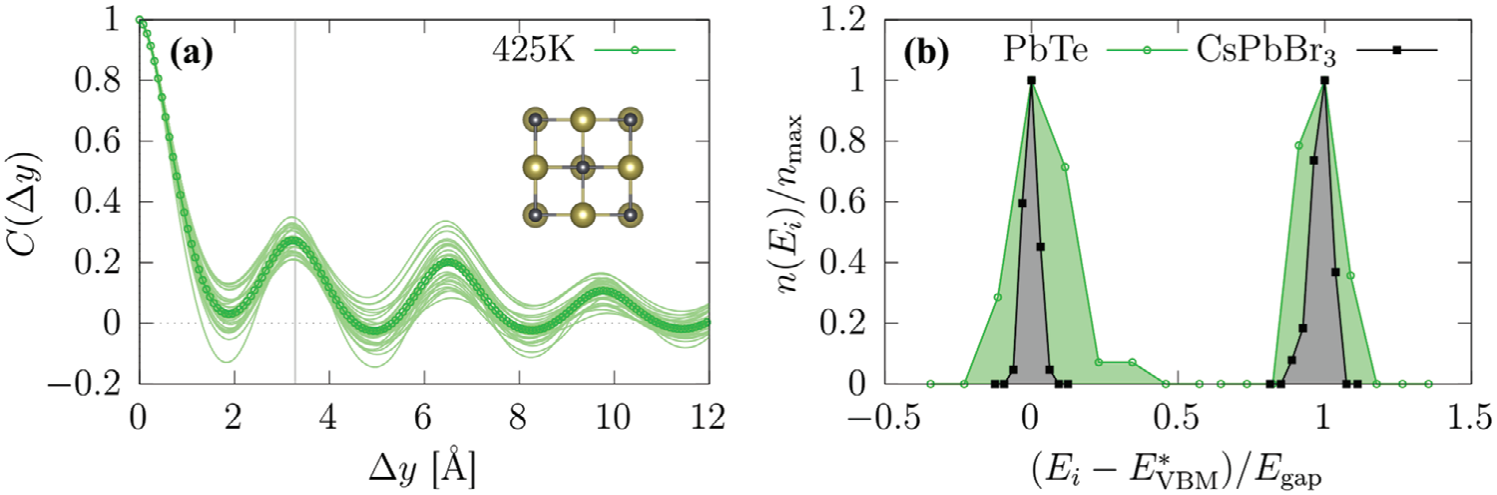
a) *C*(Δ*y*) for PbTe material calculated at 425 K; the inset depicts a structural representation of this system and the gray vertical line indicates the nominal Pb‐Te bonding distance. b) Normalized histograms of the band‐edge energies calculated along the MD trajectories of CsPbBr_3_ (black curve) and PbTe (green curve) at 425 K. The histograms were centered to the VBM and conduction band minimum (CBM) energy with highest occurrence, $E_{\mathrm{VBM}/\mathrm{CBM}}^{*}$, in order to allow for a direct comparison of the band‐edge distributions of CsPbBr_3_ and PbTe (see Methods Section).

Our findings suggest that the dynamic structural flexibility of HaPs, such as CsPbBr_3_ with its apparent degree of transversality, may play an active role in determining the favorable optoelectronic properties of HaPs, because a short‐ranged disorder potential implies sharper optical absorption onsets. To test this hypothesis, we consider PbTe as a counter‐example for a material sharing many key properties with CsPbBr_3_ (see Supporting Information for a detailed discussion): bonding properties including the anti‐bonding nature of the valence band maximum (VBM) formed by σ interactions of anion p and cation s orbitals, the cross‐gap hybridization of the anion p orbitals, a stereochemically active lone pair of electrons^[^ ^71^, ^72^, ^73^ ^]^ as well as vibrational anharmonicities^[^ ^38^, ^39^ ^]^ and, interestingly, a similar type of resonant bonding mechanism.^[^ ^67^ ^]^ Importantly, however, given its rocksalt structure (see inset in **Figure** **5**a), from symmetry considerations PbTe cannot exhibit any degree of transversality. As a consequence of the absence of transversality, any atomic displacement must perturb the resonant network to a certain degree, which leads to longer‐ranged changes in the charge‐density response to atomic displacements, as we explicitly show in the Supporting Information. In full agreement with the expectations borne from this reasoning, we find features in *C*(Δ*y*) for PbTe (see Figure 5a) reaching beyond several unit cells when we perform MD at 425 K, which is in sharp contrast to the ultra short‐ranged correlations found in CsPbBr_3_ (*cf*., Figure 4a); note that the oscillatory features can be explained by thermal noise that is due to the atomic displacements modulating the crystal periodic potential (see Supporting Information). Most importantly, comparing the conduction and valence band histograms of PbTe and CsPbBr_3_ (see Figure 5b) shows that the absence of transversality leads to much broader distributions, implying a larger amount of thermally‐induced tail‐states and energetic disorder and, thus, less sharp optical absorption edges and larger Urbach energies. We therefore speculate that the much larger Urbach energy of PbTe^[^ ^74^ ^]^ compared to CsPbBr_3_^[^ ^75^ ^]^ could in part be due to its more long‐ranged disorder correlations that we have demonstrated here to be a direct consequence of the absence of transversality, keeping in mind that other extrinsic factors may impact the Urbach energy of HaPs too.^[^ ^61^ ^]^ Altogether, these findings demonstrate that the dynamic structural flexibility of CsPbBr_3_ that we characterized here by the large degree of transversality in the Br displacements, potentially plays an important role in establishing the favorable optoelectronic properties of HaPs.

Finally, we would like to put our work in context of recent findings in the literature. Several recent studies reported an important role of specific vibrational modes that are very similar or at least closely related to the transversal modes we highlight here: for example, the 2D overdamped fluctuations recently found in CsPbBr_3_^[^ ^22^ ^]^ as well as the X–Pb–X scissoring mode.^[^ ^76^ ^]^ both of which have also been discussed to imply certain dynamic modulations to the band gap.^[^ ^22^, ^76^ ^]^ Another case in point that these modes are relevant for the optoelectronic properties of HaPs is that their counterparts, that is, the longitudinal modes, were previously found to be involved in so‐called cage vibrations that were discussed to be the main finite‐temperature contributors to the Urbach energy.^[^ ^77^ ^]^ This assessment is in full agreement with the expectations borne from our findings that disorder correlations associated with longitudinal displacements would be longer ranged (*cf*., Figure 4). Therefore, we believe that our findings motivate a wider investigation of how the dynamic structural flexibility in HaPs and related materials could be exploited toward design of materials with improved properties.

## 3 Conclusion

In summary, we investigated the effect of the dynamic structural flexibility of HaPs on their optoelectronic properties by means of first‐principles MD for the prototypical model system CsPbBr_3_ in the cubic phase. We found that the vibrations in the HaP lattice feature a distinctive property of transversality, which describes the large displacements of halide ions along directions that are orthogonal to the Pb–Br–Pb bonding axis. It was demonstrated that this property leads to a rapid rise of the JDOS that is concurrent with a shortening of disorder correlations, which implies a sharpening of the distribution of band edges, a reduction of the number of thermally induced tail‐states and, by extension, a sharper optical absorption profile. Since the related Urbach tail determines the losses of open‐circuit voltage in the radiative limit,^[^ ^78^ ^]^ this finding is potentially relevant for the design of solar materials. We have also contrasted these findings to the case of PbTe, a similarly anharmonic material also exhibiting resonant bonding and a similar electronic structure, but lacking the dynamic structural flexibility of the perovskite lattice as expressed in transversality. Our findings establish the important link between dynamic structural flexibility of HaPs with their favorable optoelectronic properties. This suggests that the unusual lattice vibrational properties of HaPs might not just be a mere coincidence and could be used to guide the material design of new compounds with similarly favorable or even enhanced optoelectronic properties.

## 4 Methods

### 4.1 Static DFT Calculations

DFT calculations were performed with the Vienna ab‐initio simulation package (VASP) code,^[^ ^79^ ^]^ using the projector‐augmented wave (PAW) method^[^ ^80^ ^]^ applying the “normal” version of the code‐supplied PAW potentials unless otherwise noted. Exchange‐correlation interactions were described using the Perdew–Burke–Ernzerhof (PBE) form of the generalized gradient approximation,^[^ ^81^ ^]^ including corrections for dispersive interactions according to the Tkatchenko–Scheffler scheme.^[^ ^82^ ^]^ This setup has been shown to allow for an accurate description of the finite‐temperature structural dynamics of HaPs.^[^ ^83^ ^]^ The ionic and lattice degrees‐of‐freedom of CsPbBr_3_ were optimized, starting from a cubic structure, resulting in a final lattice constant of 5.81 Å. For PbTe, a conventional rocksalt unit cell (8 atoms), obtained from the Crystallography Open Database,^[^ ^84^ ^]^ (COD‐ID: 9008696)^[^ ^85^ ^]^ was used and it was relaxed to a lattice constant of 6.56 Å. For structural relaxation and static DFT calculations, an energy convergence threshold of 10^−8^ eV, a Γ‐centered *k*‐grid of 6 × 6 × 6 and a plane‐wave cutoff energy of 500 eV (CsPbBr_3_) and 375 eV (PbTe) were used. The geometries of CsPbbr_3_ and PbTe were optimized until residual forces were below 10^−3^ eVÅ ^−1^.

### 4.2 First‐Principles MD Calculations

DFT‐MD simulations were performed on a 4 × 4 × 2 (160 atoms) supercell of CsPbBr_3_ and a 3 × 3 × 2 (144 atoms) of PbTe, using the canonical (NVT) ensemble at *T* = 425 K with a Nosé–Hoover thermostat as implemented in the VASP code.^[^ ^86^ ^]^ The simulation timestep was set to 8 fs for CsPbBr_3_ and 10 fs for PbTe. For the sake of efficiency, different settings for the self‐consistent calculations in each ionic step were employed: the “GW” PAW potentials were used, together with plane‐wave cutoffs of 250 eV (CsPbBr_3_) and 240 eV (PbTe), a single *k*‐point and an energy convergence threshold of 10^−6^ eV. The system was equilibrated for 5 ps and a subsequent trajectory of 150 ps was used for analysis.

### 4.3 JDOS, Disorder Potentials, and Band‐Edge Histograms

JDOS, disorder potential, and band‐edge histograms were calculated from snapshots of instantaneous configurations along the MD trajectory. The charge density and electrostatic potentials were then computed using a 1 × 1 × 2 *k*‐grid for CsPbBr_3_ and 2 × 2 × 3 *k*‐grid for PbTe, preserving the density of *k*‐points in the supercell as compared with the unit‐cell calculations. To obtain the JDOS, non‐selfconsistent calculations were subsequently performed on a finer, 3 × 3 × 6 *k*‐grid, for 90 snapshots that were evenly distributed along the trajectory and had filtered out those structures whose geometries resulted in band‐gap energy lower than 80% of the average value. The JDOS was then calculated from the eigenvalues, $\varepsilon_{i}\left(k\right)$, as^[^ ^87^ ^]^

(2) $$j\left(E\right)=2\sum_{v,c,k}\frac{w_{k}}{\sigma \sqrt{2\pi }}e^{-\frac{{\left(\varepsilon_{c}\left(k\right)-\varepsilon_{v}\left(k\right)-E\right)}^{2}}{2\sigma^{2}}}$$

where the *v* and *c* indices signify valence and conduction bands, respectively, k the *k*‐points with their respective weighting $w_{k}$, and σ the spectral broadening that was set to 20 meV.

The autocorrelation, *C*(Δ*y*), was calculated—as detailed in a previous work—as the average of the instantaneous autocorrelations^[^ ^33^ ^]^

(3) $$C_{i}\left(\Delta y\right)=\frac{\left\langle \Delta V_{i}\left(y+\Delta y\right)\cdot \Delta V_{i}\left(y\right)\right\rangle }{\left\langle \Delta V_{i}\left(y\right)\cdot \Delta V_{i}\left(y\right)\right\rangle }$$

where Δ*V*_*i*_ is the *xz*‐averaged change in the electrostatic potential of configuration *i* with respect to the average potential along the trajectory, and *C*(Δ*y*) is averaged over *N* = 30 instantaneous configurations. To separate the effect of longitudinal and transversal Br displacements in the calculations of *C*(Δ*y*), the MD trajectory was modified as follows: for each Br, the *x*, *y*, and *z* Cartesian components of a given displacement were separated into longitudinal and transversal contributions (*cf*., Figure 2a), setting either one of them as well as Cs and Pb displacements to zero. The CBM and VBM distributions in Figure 5b were calculated using the respective eigenvalues obtained from DFT calculations performed for 90 configurations along the MD trajectory. These histograms were centered to the VBM/CBM energy with highest occurrence, $E_{\mathrm{VBM}/\mathrm{CBM}}^{*}$, using $E_{\mathrm{gap}}^{*}=E_{\mathrm{CBM}}^{*}-E_{\mathrm{VBM}}^{*}$ normalizing to the respective count, that is $n_{\max}=n\left(E_{\mathrm{VBM}/\mathrm{CBM}}^{*}\right)$. The normalization allows for a direct comparison of the band‐edge distributions of CsPbBr_3_ and PbTe despite their different values of the band gap.

## Conflict of Interest

The authors declare no conflict of interest.

## Supporting information

Supporting Information

## Data Availability

The data that support the findings of this study are available from the corresponding author upon reasonable request.
