# Supplementary material for: Transversal Halide Motion Intensifies Band‐To‐Band Transitions in Halide Perovskites
Source: Adv Sci (Weinh). 2022 Apr 4;9(16):2200706. doi: 10.1002/advs.202200706 (PMC9165501; doi:10.1002/advs.202200706)
Supplement: Supplementary file 1 — Supporting Information [file ADVS-9-2200706-s001.pdf]

ADVANCED SCIENCE
Open Access

Supporting Information

for *Adv. Sci.*, DOI 10.1002/advs.202200706

Transversal Halide Motion Intensifies Band-To-Band Transitions in Halide Perovskites

*Christian Gehrmann, Sebastián Caicedo-Dávila, Xiangzhou Zhu and David A. Egger**

Supporting Information: Transversal Halide Motion Intensifies Band-To-Band Transitions in Halide Perovskites

Christian Gehrmann, Sebastián Caicedo-Dávila, Xiangzhou Zhu, and David A. Egger

Department of Physics, Technical University of Munich, Garching, Germany

Contents

1 Relation between Br displacement components and octahedral rotation 2

2 Occurrence and energetics of Br motion components 4

3 Electronic and vibrational properties of PbTe 6

4 Toy model for oscillatory correlations in PbTe 9

5 References 11

1 Relation between Br displacement components and octahedral rotation

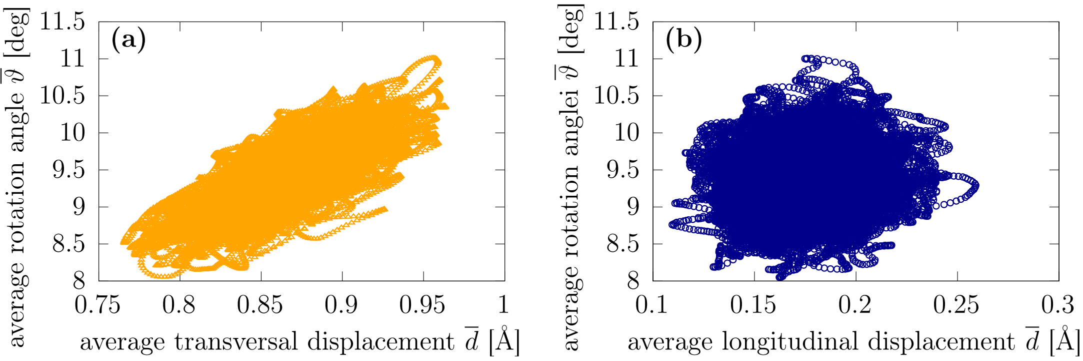

Figure 1: **(a)** Average octahedral rotation angle $\overline{\vartheta}$ as a function of the average transversal displacement. A clear correlation between displacement and rotation angle can be seen. **(b)** Same as (a), but now for the average longitudinal displacement. No clear correlation between rotation angle and displacement can be seen. The average transversal / longitudinal displacements were calculated as $\overline{d}^{\text{transv/longi}}(t) = N_{\text{Br}}^{-1} \sum_{i=1}^{N_{\text{Br}}} d_i^{\text{transv/longi}}(t)$. For calculation of the rotation angle, see Fig. 2. We expect that transversal and longitudinal displacements not manifesting as octahedral rotation to contribute to octahedral deformation.

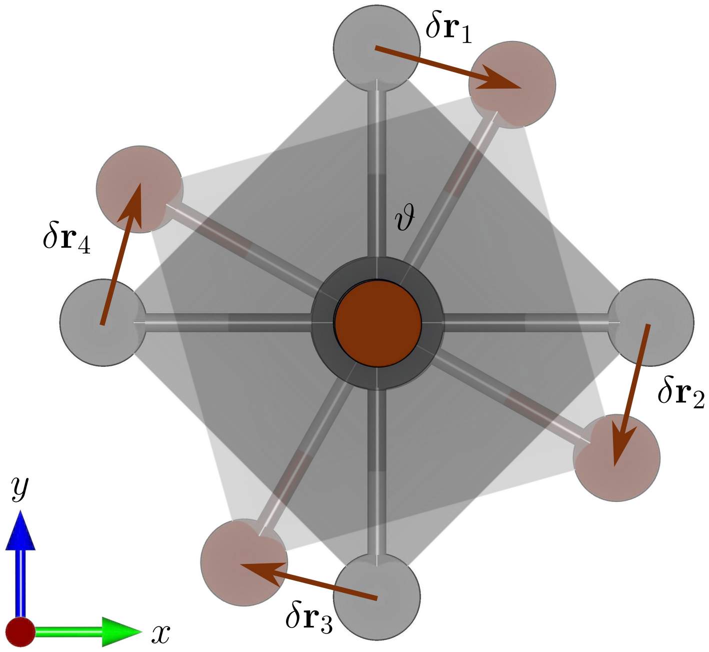

Figure 2: The octahedral rotation angle ϑ in the $x-y$ plane (shown schematically by two octahedra that are colored differently) is calculated from the following displacements of the 4 Br atoms: $\delta\mathbf{r}_1 = d_{\text{Pb-Br}}(\sin\vartheta_1, 1-\cos\vartheta_1)^T$, $\delta\mathbf{r}_2 = d_{\text{Pb-Br}}(1-\cos\vartheta_2, -\sin\vartheta_2)^T$, $\delta\mathbf{r}_3 = d_{\text{Pb-Br}}(-\sin\vartheta_3, \cos\vartheta_3 - 1)^T$, and $\delta\mathbf{r}_4 = d_{\text{Pb-Br}}(\cos\vartheta_4 - 1, \sin\vartheta_4)^T$. Here, $d_{\text{Pb-Br}}$ is the Pb-Br distance. From this we obtain: $\vartheta = \arcsin\left(\frac{1}{4}\sum_{i=1}^{4}\sin\vartheta_i\right) = \arcsin\left(\frac{1}{4d_{\text{Pb-Br}}}[\delta x_1 - \delta y_2 - \delta x_3 + \delta y_4]\right)$. The rotation angles in $x-z$ and $y-z$ plane can be calculated equivalently to then obtain the average angle, $\overline{\vartheta}$ (averaged across all octahedra and the 3 planes).

2 Occurrence and energetics of Br motion components

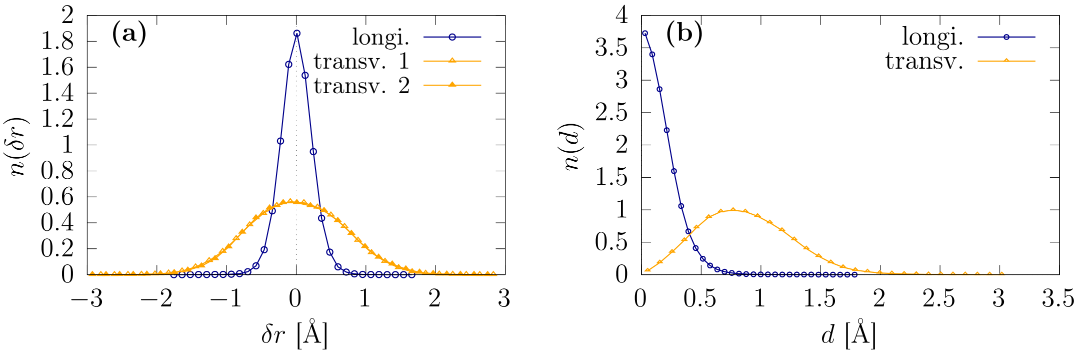

Figure 3: **(a)** Normalized histograms for the displacements $\delta r_i^{\mathrm{longi/transv}}$ along one longitudinal and two transversal components; the latter two curves are on top of each other. **(b)** Normalized histograms for the norms $d^{\mathrm{longi/transv}} = \|\delta\mathbf{r}^{\mathrm{longi/transv}}\|$ of longitudinal and transversal displacements. Note that they are normalized such that they integrate to 1. Both analyses show that transversal displacements feature larger magnitudes and broader distributions, and that they are thus more likely to enter the anharmonic regime of the potential energy surface.

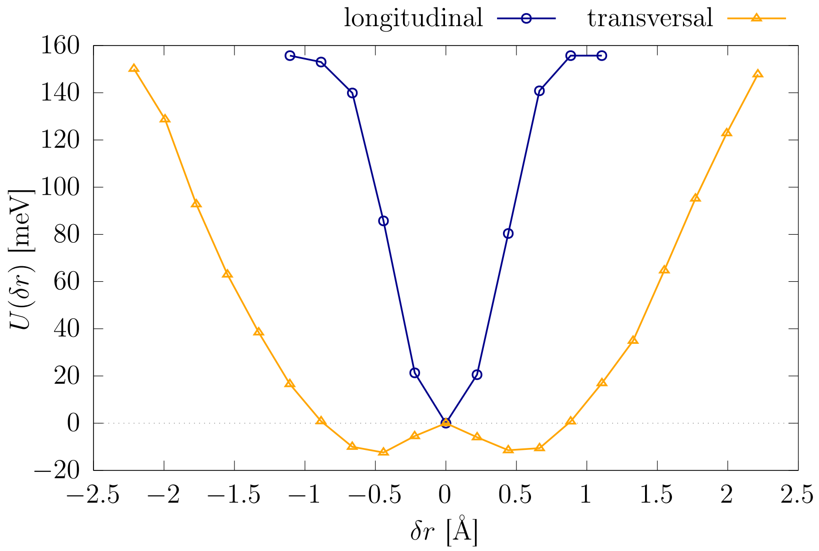

Figure 4: Potential energy, $U(\delta r)$, as a function of the atomic displacement, δr, calculated for one longitudinal and one transversal component of the Br displacements. The potential is obtained from inversion of a histogram of displacements, $n(\delta r_1, \delta r_2, \delta r_3)$, using Boltzmann statistics, since $n(\delta r_1, \delta r_2, \delta r_3) \propto \exp\{-U(\delta r_1, \delta r_2, \delta r_3)/k_B T\}$. Consequently, the the potential can be obtained as: $U(\delta r_1, \delta r_2, \delta r_3) = -\ln\{n(\delta r_1, \delta r_2, \delta r_3)\} \cdot k_B T + const.$ [1] From this, it can be seen that 1. transversal displacement follow an anharmonic potential energy surface, and 2. large displacements are energetically less expensive along the transversal compared to the longitudinal direction. It is noted that unlike the distribution of displacements, where the additional components can be integrated out, i.e., $n(\delta r_1) = \iint d\delta r_2\, d\delta r_3 n(\delta r_1, \delta r_2, \delta r_3)$, the potential is not additive. Thus, we show the potential energy curves along one exemplary transversal and the longitudinal direction with the other directions kept at 0, i.e., $U(\delta r_1) = U(\delta r_1, \delta r_2 = 0, \delta r_3 = 0) = -\ln\{n(\delta r_1, \delta r_2 = 0, \delta r_3 = 0)\} \cdot k_B T + const.$

3 Electronic and vibrational properties of PbTe

In the main text we have contrasted our findings for halide perovskites (HaPs) with the material PbTe, a low band-gap semiconductor with a rocksalt crystal structure and a high thermoelectric figure of merit.[2, 3, 4, 5, 6, 7] Comparing these materials is motivated by the fact that they both exhibit relatively low phonon energies, as summarized in Table 1 and reported, for instance, in references [8, 9, 10, 11]. In Fig. 5 we compare the vibrational density of states (vdos) of PbTe as obtained from harmonic calculations, using finite displacements (black line), to the one obtained from MD simulations by means of the velocity autocorrelation function (vacf). The result of Fig. 5 confirms the low-frequency nature of the vibrations in PbTe and demonstrates the impact of anharmonicity on the lattice vibrations of this system, which is known in the literature [12, 13, 14, 15] and HaPs. [16, 17, 18, 19, 20, 21, 22, 23, 24, 25] That HaPs and PbTe share similarly important anharmonic effects can also be seen considering their similarly low phonon lifetimes of less than 10 ps, see Table 1.

The role of anharmonicity in both materials has been related to the similar bonding mechanism active in both cases. Specifically, a mechanism called resonant bonding has been discussed for the case of PbTe [12] and HaPs [26, 27, 9]. Along with this, the bonding and electronic structure of PbTe and $CsPbBr_3$ share several important aspects: like HaPs, PbTe also exhibits a stereochemically active lone pair of electrons (LPE), resulting from the hybridization of Pb-6s and Te-5p orbitals,[28] analogous to the LPE in HaPs that is resulting from Pb-6s and X-p hybridization.[29, 30] Additionally, the valence band in PbTe is formed mostly from Te-5p orbitals (that are analogous to the X-p orbitals in HaPs), while the conduction band is dominated by Pb-6p orbitals (akin to the case HaPs).[31, 32, 33, 2] The frontier part of the valence band, in both PbTe and $CsPbBr_3$, is of anti-bonding nature, formed by σ interactions.[33, 2, 31] However, the different crystal structures imply differences in the bonding nature of the conduction band: while in PbTe it is formed by σ interactions between p orbitals,[2] it has been described that in HaPs it has a larger π character[33].

While these characteristics render PbTe an interesting material as far as the importance of (anharmonic) structural dynamics and resonant bonding are concerned, there are also important differences between PbTe and HaPs. First and foremost, the crystal structure is different, with the rocksalt PbTe lacking the ability to feature any transversality, and hence being less flexible in its structural dynamics. Specifically, while both materials exhibit the same coordination for Pb, the coordination of Te is higher than that of the halides (Br in $CsPbBr_3$). We think this is essential for the structural flexibility and its consequences on band-edge distributions, as discussed in the main text. Concurrent with this are the exclusively long-ranged density responses to small finite displacements in PbTe, shown in Fig. 5. Another difference between the two materials, already mentioned in the main text, is the magnitude of the respective Urbach energy that quantifies the steepness of the optical absorption coefficient. While they have

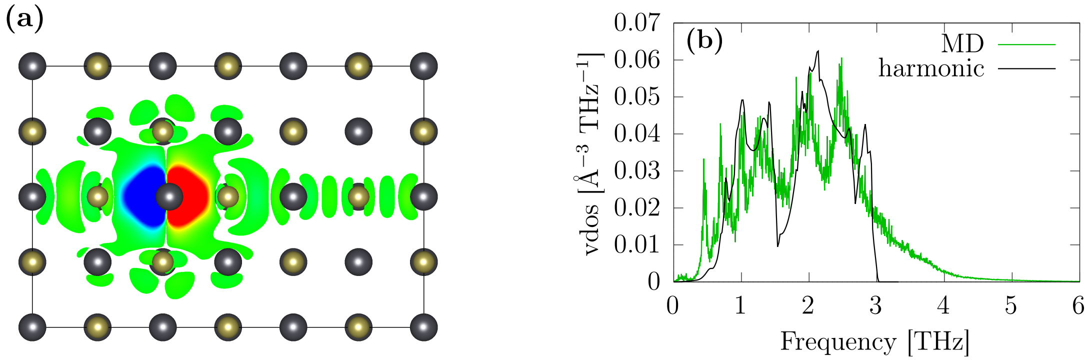

Figure 5: **(a)** Iso-surface representation (iso-level $> 8.1 \times 10^{-3}$ eÅ^{-3}) of the charge-density difference that is induced upon a displacement of one Pb atom by 5% of the primitive lattice constant in PbTe. The long-range nature indicates the presence of the resonant bonding mechanism as discussed in the main text. **(b)** Vibrational density of states (vdos) of rocksalt PbTe, as obtained from harmonic calculations using finite differences (black line) and from a Fourier transform of the MD-calculated velocity autocorrelation function (green curve). The harmonic vdos was obtained using the phonopy package [49], a 2x2x2 supercell, and a 3x3x3 Γ-centered k-point grid.

been determined to be low for HaPs, *e.g.*, approximately 15 meV in the paradigmatic $MAPbI_3$ [34, 35, 36, 37, 38] and $\lesssim$19 meV in $CsPbBr_3$ around room temperature [39]. In contrast, PbTe shows higher Urbach energies, *e.g.*, 56 meV in nanocrystalline PbTe have been reported [40]. These differences, complemented by the similarities described earlier, are considered significant for understanding the relation between structural flexibility and band-edge distributions investigated in the main text. Last but not least, PbTe is a small band-gap semiconductor with a direct band gap of around 0.3 eV located at the L-point of the Brillouin zone [41, 42, 43, 40]. In contrast, the band gap of $CsPbBr_3$ is measured to be around 2.3 eV, depending on structural phase, temperature and details in the methododology [44, 45, 46, 47, 48].

Table 1: A comparison of several properties between the two materials PbTe and $CsPbBr_3$.

	PbTe	$CsPbBr_3$
crystal structure	rocksalt	perovskite
phonon frequencies	$\lesssim$ 4 THz$^+$ [10, 11]	$\lesssim$ 5 THz [8, 9]
anharmonic	✓	✓
phonon lifetimes	$<$ 10 ps [50]	$<$ 10 ps [9, 51]
resonant bonding	✓	✓
VBM character	σ^*	σ^*
CBM character	σ^*	$\sigma^*\pi^*\pi^*$
band gap	$\sim$ 0.32 eV [41, 42, 43, 40]	2.25 - 2.38 eV§ [44, 45, 46, 47, 48]
Urbach energy	$\sim$ 56 meV† [40]	$\sim$15 meV‡ [34, 35, 36, 37, 38, 39]

$^+$see also Fig. 5

§depending on structural phase, temperature and method

†nanocrystalline, not single crystalline

‡most for $MAPbI_3$, only [39] for $CsPbBr_3$

4 Toy model for oscillatory correlations in PbTe

The correlation function of the disorder potential for the case of PbTe (Fig. 3a of the main text) shows an oscillatory behaviour and a long-range decay. Furthermore, the periodicity of the oscillatory feature coincides with the atomic distances. In order to investigate these features we investigated the correlations of a simple toy model, in which a simple one-dimensional disorder potential that preserves the translational invariance of the crystal is defined as

$$\Delta V_i(y) = A_i \sin(\omega_i\, y) \ , \tag{1}$$

with A_i determining an amplitude for the disorder potential and ω_i providing the spatial periodicity. It is shown in Fig. 6a for the simple case of $A = 1$ and $\omega = 1$. The autocorrelation function of this disorder potential

$$C_i(\Delta y) = \frac{\langle \Delta V_i(y + \Delta y) \cdot \Delta V_i(y) \rangle}{\langle \Delta V_i(y) \cdot \Delta V_i(y) \rangle} \ , \tag{2}$$

(see also eq. (2) of the main text) is again periodic, see Fig. 6b. However, due to the nuclei motion at finite temperature, the ideal periodicity is broken. We now model the breaking of periodicity occurring locally in the structure due to atomic motions at finite temperature using a disorder potential that is modulated by the atomic displacements, using randomized A_i and ω_i in Eq. (1). As can be seen in Fig. 6d, the autocorrelation function of each specific choice for A_i and ω_i (the corresponding disorder potentials are shown in Fig. 6c) remains oscillatory, but importantly also non-decaying (light red curves). However, a combination of *several modulated disorder potentials* results in a *oscillatory and decaying* autocorrelation function (dark red line in Fig. 6d). From this analysis, we conclude that the shape of the correlation function we have observed for PbTe stems from the thermal noise that is due to the atomic displacements and modulates the underlying periodic potential of the system.

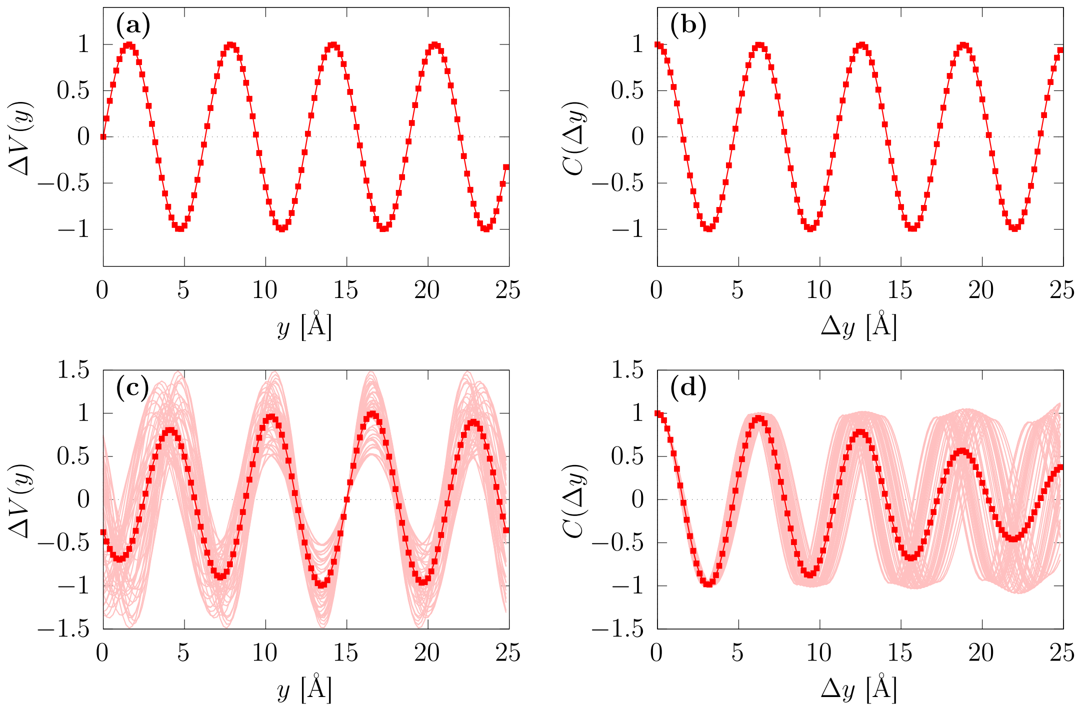

Figure 6: Panel **(a)** shows the disorder potential, Eq. (1), for $A = 1$ and $\omega = 1$. Panel **(b)** shows the autocorrelation function, as defined in Eq. (2), for the disorder potential shown in panel a. This autocorrelation is oscillatory, like the disorder potential, but does not decay. Panel **(c)** shows in light red $N = 50$ disorder potentials according to Eq. (1), with $0.5 \leq A_i \leq 1.5$ and $0.9 \leq \omega \leq 1.1$ chosen randomly. The dark-red line shows the average disorder potential $\overline{\Delta V}(\Delta y) = N^{-1}\sum_{i=1}^{N} V_i(y)$. Panel **(d)** shows in light red the autocorrelation function, as defined in Eq. (2),for the $N = 50$ disorder potentials shown in panel c. The dark red line shows the average $\overline{C}(\Delta y) = N^{-1}\sum_{i=1}^{N} C_i(\Delta y)$. The latter is oscillatory and decays for larger distances Δy, which is qualitatively similar as to what has been observed for PbTe in Fig. 3a of the main text.

5 References

[1] J. Lahnsteiner, G. Kresse, J. Heinen, M. Bokdam, *Phys. Rev. Mater.* **2018**, *2* 073604.

[2] M. K. Brod, M. Y. Toriyama, G. J. Snyder, *Chem. Mater.* **2020**, *32*, 22 9771.

[3] Y. Pei, H. Wang, G. J. Snyder, *Adv. Mater.* **2012**, *24*, 46 6125.

[4] J. Sootsman, D. Chung, M. Kanatzidis, *Angew. Chem. Int. Ed.* **2009**, *48*, 46 8616.

[5] G. Chen, M. S. Dresselhaus, G. Dresselhaus, J.-P. Fleurial, T. Caillat, *Inter. Mater. Rev.* **2003**, *48*, 1 45.

[6] K. Biswas, J. He, I. D. Blum, C.-I. Wu, T. P. Hogan, D. N. Seidman, V. P. Dravid, M. G. Kanatzidis, *Nature* **2012**, *489*, 7416 414.

[7] J. P. Heremans, V. Jovovic, E. S. Toberer, A. Saramat, K. Kurosaki, A. Charoenphakdee, S. Yamanaka, G. J. Snyder, *Science* **2008**, *321*, 5888 554.

[8] P. Guo, Y. Xia, J. Gong, C. C. Stoumpos, K. M. McCall, G. C. B. Alexander, Z. Ma, H. Zhou, D. J. Gosztola, J. B. Ketterson, M. G. Kanatzidis, T. Xu, M. K. Y. Chan, R. D. Schaller, *ACS Energy Lett.* **2017**, *2*, 10 2463.

[9] C. Gehrmann, D. A. Egger, *Nat. Commun.* **2019**, *10*, 1 3141.

[10] J. An, A. Subedi, D. Singh, *Solid State Commun.* **2008**, *148*, 9-10 417.

[11] B. Sangiorgio, E. S. Bozin, C. D. Malliakas, M. Fechner, A. Simonov, M. G. Kanatzidis, S. J. L. Billinge, N. A. Spaldin, T. Weber, *Phys. Rev. Materials* **2018**, *2*, 8.

[12] S. Lee, K. Esfarjani, T. Luo, J. Zhou, Z. Tian, G. Chen, *Nat. Commun.* **2014**, *5*, 1 3525.

[13] G. A. S. Ribeiro, L. Paulatto, R. Bianco, I. Errea, F. Mauri, M. Calandra, *Phys. Rev. B* **2018**, *97*, 1.

[14] O. Delaire, J. Ma, K. Marty, A. F. May, M. A. McGuire, M.-H. Du, D. J. Singh, A. Podlesnyak, G. Ehlers, M. D. Lumsden, B. C. Sales, *Nat. Mater.* **2011**, *10*, 8 614.

[15] A. H. Romero, E. K. U. Gross, M. J. Verstraete, O. Hellman, *Phys. Rev. B* **2015**, *91*, 21 214310.

[16] L. D. Whalley, J. M. Skelton, J. M. Frost, A. Walsh, *Phys. Rev. B* **2016**, *94*, 22 1.

[17] A. N. Beecher, O. E. Semonin, J. M. Skelton, J. M. Frost, M. W. Terban, H. Zhai, A. Alatas, J. S. Owen, A. Walsh, S. J. Billinge, *ACS Energy Lett.* **2016**, *1*, 4 880.

[18] O. Yaffe, Y. Guo, L. Z. Tan, D. A. Egger, T. Hull, C. C. Stoumpos, F. Zheng, T. F. Heinz, L. Kronik, M. G. Kanatzidis, J. S. Owen, A. M. Rappe, M. A. Pimenta, L. E. Brus, *Phys. Rev. Lett.* **2017**, *118*, 13 1.

[19] M. A. Carignano, S. A. Aravindh, I. S. Roqan, J. Even, C. Katan, *J. Phys. Chem. C* **2017**, *121*, 38 20729.

[20] A. Marronnier, H. Lee, B. Geffroy, J. Even, Y. Bonnassieux, G. Roma, *J. Phys. Chem. Lett.* **2017**, *8*, 12 2659.

[21] A. Gold-Parker, P. M. Gehring, J. M. Skelton, I. C. Smith, D. Parshall, J. M. Frost, H. I. Karunadasa, A. Walsh, M. F. Toney, *Proc. Natl. Acad. Sci. U. S. A.* **2018**, *115*, 47 11905.

[22] A. Marronnier, G. Roma, S. Boyer-Richard, L. Pedesseau, J. M. Jancu, Y. Bonnassieux, C. Katan, C. C. Stoumpos, M. G. Kanatzidis, J. Even, *ACS Nano* **2018**, *12*, 4 3477.

[23] T. Zhu, E. Ertekin, *Energy Environ. Sci.* **2019**, *12*, 1 216.

[24] R. Sharma, Z. Dai, L. Gao, T. M. Brenner, L. Yadgarov, J. Zhang, Y. Rakita, R. Korobko, A. M. Rappe, O. Yaffe, *Phys. Rev. Mater.* **2020**, *4*, 9 1.

[25] J. Klarbring, O. Hellman, I. A. Abrikosov, S. I. Simak, *Phys. Rev. Lett.* **2020**, *125*, 4 45701.

[26] D. Weber, *Z. Naturforsch. B* **1978**, *33*, 12 1443.

[27] T. Zhu, E. Ertekin, *Energy Environ. Sci.* **2019**, *12*, 1 216.

[28] S. H. Wei, A. Zunger, *Phys. Rev. B* **1997**, *55*, 20 13605.

[29] D. H. Fabini, R. Seshadri, M. G. Kanatzidis, *MRS Bull.* **2020**, *45*, 6 467.

[30] L. Gao, L. Yadgarov, R. Sharma, R. Korobko, K. M. McCall, D. H. Fabini, C. C. Stoumpos, M. G. Kanatzidis, A. M. Rappe, O. Yaffe, *Mater. Adv.* **2021**.

[31] U. V. Waghmare, N. A. Spaldin, H. C. Kandpal, R. Seshadri, *Phys. Rev. B* **2003**, *67*, 12 10.

[32] K. Xiong, G. Lee, R. P. Gupta, W. Wang, B. E. Gnade, K. Cho, *J. Phys. D. Appl. Phys.* **2010**, *43*, 40.

[33] M. G. Goesten, R. Hoffmann, *J. Am. Chem. Soc.* **2018**, *140*, 40 12996.

[34] A. Sadhanala, F. Deschler, T. H. Thomas, S. E. Dutton, K. C. Goedel, F. C. Hanusch, M. L. Lai, U. Steiner, T. Bein, P. Docampo, D. Cahen, R. H. Friend, *J. Phys. Chem. Lett.* **2014**, *5*, 15 2501.

[35] S. De Wolf, J. Holovsky, S.-J. Moon, P. Lper, B. Niesen, M. Ledinsky, F.-J. Haug, J.-H. Yum, C. Ballif, *J. Phys. Chem. Lett.* **2014**, *5*, 6 1035.

[36] S. P. Senanayak, B. Yang, T. H. Thomas, N. Giesbrecht, W. Huang, E. Gann, B. Nair, K. Goedel, S. Guha, X. Moya, C. R. McNeill, P. Docampo, A. Sadhanala, R. H. Friend, H. Sirringhaus, *Sci. Adv.* **2017**, *3*, 1 e1601935.

[37] J. B. Patel, Q. Lin, O. Zadvorna, C. L. Davies, L. M. Herz, M. B. Johnston, *J. Phys. Chem. Lett.* **2018**, *9*, 1 263.

[38] M. Ledinsky, T. Schnfeldov, J. Holovsk, E. Aydin, Z. Hjkov, L. Landov, N. Neykov, A. Fejfar, S. De Wolf, *J. Phys. Chem. Lett.* **2019**, *10*, 6 1368.

[39] Y. Rakita, N. Kedem, S. Gupta, A. Sadhanala, V. Kalchenko, M. L. Bhm, M. Kulbak, R. H. Friend, D. Cahen, G. Hodes, *Crys. Growth Des.* **2016**, *16*, 10 5717.

[40] J. Wang, J. Hu, X. Sun, A. M. Agarwal, L. C. Kimerling, D. R. Lim, R. A. Synowicki, *J. Appl. Phys.* **2008**, *104*, 5 053707.

[41] W. W. Scanlon, *J. Phys. Chem. Solids* **1959**, *8* 423.

[42] B. Gelmont, T. Globus, A. Matveenko, *Solid State Commun.* **1981**, *38*, 10 931.

[43] R. Dalven, *Infrared Physics* **1969**, *9*, 4 141.

[44] L. Lang, Y.-Y. Zhang, P. Xu, S. Chen, H. J. Xiang, X. G. Gong, *Phys. Rev. B* **2015**, *92*, 7 075102.

[45] M. Kulbak, D. Cahen, G. Hodes, *J. Phys. Chem. Lett.* **2015**, *6*, 13 2452.

[46] C. C. Stoumpos, C. D. Malliakas, J. A. Peters, Z. Liu, M. Sebastian, J. Im, T. C. Chasapis, A. C. Wibowo, D. Y. Chung, A. J. Freeman, B. W. Wessels, M. G. Kanatzidis, *Crys. Growth Des.* **2013**, *13*, 7 2722.

[47] J. Lei, F. Gao, H. Wang, J. Li, J. Jiang, X. Wu, R. Gao, Z. Yang, S. F. Liu, *Sol. Energy Mater. Sol. Cells* **2018**, *187* 1.

[48] G. Mannino, I. Deretzis, E. Smecca, A. La Magna, A. Alberti, D. Ceratti, D. Cahen, *J. Phys. Chem. Lett.* **2020**, *11*, 7 2490.

[49] A. Togo, I. Tanaka, *Scr. Mater.* **2015**, *108* 1.

[50] Y. Lu, T. Sun, D.-B. Zhang, *Phys. Rev. B* **2018**, *97*, 17 174304.

[51] J. Lahnsteiner, M. Bokdam, *arXiv Preprint* **2021**, arXiv: 2101.06099 [cond-mat].
